# Hyponatremia: Is it related to the seasons?

**DOI:** 10.5937/jomb0-30409

**Published:** 2021-09-03

**Authors:** Atila Altuntas

**Affiliations:** 1 Suleyman Demirel University, Faculty of Medicine, Department of Internal Medicine, Division of Nephrology, Isparta, Turkey

**Keywords:** hyponatremia, season, humidity, temperature, renal failure, hiponatremija, godišnje doba, vlažnost vazduha, temperatura, bubrežna insuficijencija

## Abstract

**Background:**

Hyponatremia is a common electrolyte disorder in inpatients related to morbidity and mortality. In this study, we aimed to examine whether there is a relationship between the incidence of hyponatremia and the seasons among the patients hospitalized in our nephrology department.

**Methods:**

The inpatients in our Nephrology Department between 2012 and 2015 were retrospectively analyzed. The patients with serum sodium levels below 135 mmol/L were included in the study. Hyponatremia incidence was calculated as the proportion of inpatients with low sodium levels in a season to the total number of inpatients in the same season.

**Results:**

Out of 1950 inpatients in four years, 509 were found to have hyponatremia (26.1%). The mean serum sodium level of the patients was 129.7±4.7 mmol/L. Hyponatremia incidences in autumn, winter, spring, and summer were found to be 28.7%, 15.4%, 20.4%, and 36.6%, respectively. Upon comparing the incidence of hyponatremia in patients hospitalized in winter and summer seasons, there was a significantly higher incidence of hyponatremia in summer (p<0.001). We found a positive correlation between hyponatremia incidence and temperature (r=0.867, p=0.001). However, there was a negative correlation between hyponatremia incidence and relative humidity (r=-0.735, p=0.001).

**Conclusions:**

The highest hyponatremia incidence was observed in summer in a four-year period. Loss of sodium by perspiration, along with increased temperature and/or excessive hypotonic fluid intake, might contribute to the development of hyponatremia.

## Abstract (sr)

**Uvod:**

Hiponatremija je uobičajen poremećaj elektrolita kod bolničkih pacijenata i u vezi je sa morbiditetom i mortalitetom. U ovom istraživanju smo imali za cilj da ispitamo postoji li veza između učestalosti hiponatremije i godišnjih doba među pacijentima hospitalizovanim na našem nefrološkom odeljenju.

**Metode:**

Retrospektivno su analizirani bolnički pacijenti na našem nefrološkom odeljenju između 2012. i 2015. godine. Pacijenti sa nivoom natrijuma u serumu ispod 135 mmol/L su bili uključeni u studiju. Učestalost hiponatremije je izračunata kao udeo stacionarnih bolesnika sa niskim nivoom natrijuma u sezoni u odnosu na ukupan broj stacionarnih bolesnika u istoj sezoni.

**Rezultati:**

Od 1950 bolničkih pacijenata u četvoro - godišnjem periodu, kod 509 je utvrđeno da ima hiponatremiju (26,1%). Srednji nivo natrijuma u serumu kod pacijenata je bio 129,7±4,7 mmol/L. Utvrđeno je da je učestalost hiponatremije u jesen, zimu, proleće i leto redom bila 28,7%, 15,4%, 20,4% i 36,6%. Upoređujući učestalost hiponatremije kod pacijenata hospitalizovanih u zimskoj i letnjoj sezoni, primećena je značajno veća učestalost hiponatremije leti (p<0,001). Pronašli smo pozitivnu korelaciju između učestalosti hiponatremije i temperature (r=0,867, p=0,001). Međutim, postojala je negativna korelacija između incidence hiponatremije i relativne vlažnosti (r=-0,735, p=0,001).

**Zaključak:**

Najveća učestalost hiponatremije je primećena leti tokom četvorogodišnjeg perioda. Gubitak natrijuma znojenjem, zajedno sa povišenom temperaturom i/ili prekomernim unosom hipotonične tečnosti može doprineti razvoju hiponatremije.

## Introduction

Hyponatremia, which is generally defined as serum sodium concentration below 135 mmol/L [1], is a common electrolyte disorder that can affect approximately 30% of inpatients in hospitals [2]. Hyponatremic patients can have up to 60-fold morbidity and mortality compared to non-hyponatremic patients [3]. Hyponatremia occurs in numerous medical conditions such as heart, kidney, liver failure and malignancies, and can develop due to the use of certain drugs [4] [5]. Water retention, or less commonly sodium loss, plays a role in the pathogenesis of hyponatremia [4].

Serum sodium abnormalities due to high air temperature are common [6]. In addition to the studies evaluating the relationship between hyponatremia and warm weather [7] [8], there are also studies revealing the relationship between hyponatremia and seasonal change in the literature [9] [10]. When the literature is reviewed, it is seen that there are conflicting results in studies on the relationship between seasons and the incidence of hyponatremia. In this study, we aimed to examine the relationship between the incidence of hyponatremia and seasonal change in inpatients in our nephrology department.

## Material and Methods

Data were retrospectively collected from the hospital records of the inpatients in the Department of Nephrology, Suleyman Demirel University Hospital of Medical Faculty, Isparta, between 2012 and 2015. The study population included patients over the age of 18 with sodium levels of <135 mmol/L. For serum sodium levels, the initial values of the patients were taken when they were admitted to the nephrology service. Volume conditions (hypovolemia, euvolemia and hypervolemia) of the patients were recorded according to physical examination findings in patient records. Accompanying diseases and medications were also recorded. Data on demographics (such as age, gender), serum sodium, blood urea nitrogen (BUN), serum creatinine values, dialysis and mortality status were collected. Beckman Coulter AU 5800 (Miami, USA) autoanalyzer and spectrophotometric method were used to conduct biochemical analyses. The number of patients with hyponatremia was recorded in each three-month period according to seasons as follows; autumn (September-November), winter (December-February), spring (March-May), and summer (June-August). Hyponatremia incidence was calculated as the proportion of inpatients with low sodium levels within a three-month period, to the total number of inpatients in the nephrology department in the same season (hyponatremia incidence (%) = (the number of hyponatremic patients in a three-month period x 100)/(the total number of inpatients in a three-month period)). Mean temperature and humidity values of seasons were obtained from Isparta Regional Directorate of Meteorology (mean temperature (°C) and mean relative humidity (%) of each month, including the lowest and the highest values). Ethics Committee approval was obtained from the local ethics committee of Süleyman Demirel University for conducting the study.

### Statistical Analysis

Statistical Package for the Social Sciences for Windows Software, version 22 (SPSS Inc., Chicago, IL, USA) was used for data analysis. Continuous variables were expressed as mean ± standard deviation (SD), and *p*-value <0.05 was considered to be statistically significant. Frequencies and percentages were used for categorical data. For the comparison of quantitative variables, the suitability of parametric test conditions was checked. For variables that met parametric test conditions, one-way analysis of variance (ANOVA) and post hoc multiple comparison tests (LSD) were performed on the data of biochemical variables to examine the significance of the differences among groups. For the evaluation of categorical variables, the chi-square (x²) test was used. Z ratio test was used for the comparison of the ratio between groups. Minitab software (Minitab Inc., 1998) was used to determine the differences between the percentage of hyponatremia incidence and season sub-groups. Pearson correlation coefficient was used to investigate the correlations between parametric variables, and Spearman correlation coefficient was used for non-parametric variables. Multiple logistic regression analysis was performed to determine the variables that had the most significant effect on the level of hyponatremia.

## Results

The patients living in the city of Isparta, located in the Southwest of Turkey with an altitude of 1035 meters, were included in the study group. Out of 1950 inpatients between 2012-2015, 509 (26.1%) were diagnosed with hyponatremia. Of these patients, 14.87% (n: 290) were elderly patients (65 years old and over) and 11.23% (n: 219) were adults (between 18 and 64 years old) (p: 0.001). The number of patient beds and healthcare personnel in nephrology inpatient service remained unchanged during that year. Mean serum sodium concentration, BUN and serum creatinine values of the patients were measured as 129.7±4.7 mmol/L, 22.4±13.8 mmol/L and 0.38±0.26 mmol/L, respectively. The mean age of the patients was 64.4±15.4, and 49.7% of the patients (n=253) were male. Serum sodium levels of the elderly and adult patient groups were similar (Na: 129±4.43, 129.48±5.02 mmol/L, p: 0.311, respectively). The mean age and gender distribution of the patients according to seasons were similar (Table 1).

**Table 1:** Clinical and demographic data of the patients according to the seasons Values for continuous variables are given as mean ± standard deviation; values for categorical variables are given as percentages. *: This *p*-value represents the significance of the 4x3 chi-square test. Abbreviations: BUN, blood urea nitrogen; BP, blood pressure; CKD, chronic kidney disease; AKI, acute kidney injury; CHF, congestive heart failure; CCB, Calcium channel blocker; ACEI, angiotensin-converting enzyme inhibitor; ARB, angiotensin receptor blocker; n (%), number and percentage of patients; ^a,b,c,d^ There is a significant difference between the parameters marked with the same letter (p<0.05).

	Autumn n = 109	Winter n = 76	Spring n = 89	Summer n = 235	*p*-value
Age (years)	65.9±10.4	63.1±18.4	62.1±12.1	65.1±17.1	0.26
Female/Male	54/55	34/42	54/35	114/121	0.16
Serum sodium (mmol/L)	129.4.±5.9	130.5±5.2	129.7±4.1	129.5±4.3	0.37
Creatinine (mmol/L)	0.36±0.23	0.35±0.21	0.43±0.33	0.38±0.27	0.15
BUN (mmol/L)	22.6±13.9	21.9±12.7	22.5±13.1	22.3±14.4	0.99
Systolic BP (mm Hg)	125.4±29.8	119.1±26.1	119.2±29.3	125±28.1	0.18
Diastolic BP (mm Hg)	74.1±16.7	73.7±15.9	71.7±13.8	76.1±17.1	0.17
Incidence of hyponatremia (%)	28.7%^a^	15.4%^a,b^	20.4%^c^	36.6%^b,c^	0.001
Existing disease, n (%)					
CKD	83 (76.1%)	58 (76.3%)	56 (62.9%)	161 (68.5%)	0.12
AKI	25 (22.9%)	15 (19.7%)	29 (32.6%)	66 (28.1%)	0.21
CHF	24 (22.0%)	15 (19.7%)	10 (11.2%)	48 (20.4%)	0.22
Nephrotic syndrome	4 (3.7%)	2 (2.6%)	8 (9.0%)	8 (3.4%)	0.12
Cirrhosis	0 (0.0%)	0 (0.0%)	0 (0.0%)	3 (1.3%)	0.32
Diabetes mellitus	59 (54.1%)	38 (50%)	35 (39.3%)	96 (40.9%)	0.07
Hypertension	74 (67.9%)	45 (59.2%)	45 (50.6%)	129 (54.9%)	0.06
Medication use, n (%)					
ACEI/ARB	31 (28.4%)	19 (25%)	16 (18.0%)	77 (32.8%)	0.06
CCB	34 (31.2%)	23 (30.3%)	15 (16.9%)	71 (30.2%)	0.08
Thiazide	16 (14.7%)	12 (15.8%)	11 (12.4%)	31 (13.2%)	0.91
Furosemide	32 (29.4%)	22 (28.9%)	18 (20.2%)	58 (24.7%)	0.44
Spironolactone	9 (8.3%)	5 (6.6%)	3 (3.4%)	19 (8.1%)	0.48
Antidepressant	15 (13.8%)	12 (15.8%)	12 (13.5%)	29 (12.3%)	0.89
Volume status					
Hypovolemic	51 (46.8%)^a,b^	60 (78.9%)^a,c^	71 (79.8%)^b,d^	143 (60.9%)^c,d^	0.001*
Euvolemic	1 (0.9%)	2 (2.6%)	2 (2.2%)	3 (1.3)	
Hypervolemic	57 (52.3%)^a,b^	14 (18.4%)^a,c^	16 (18%)^b,d^	89 (37.9%)^c,d^	

**Figure 1:**
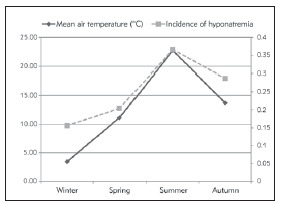
Incidence of hyponatremia, percent (%) related to seasonal variability of temperature

**Figure 2:**
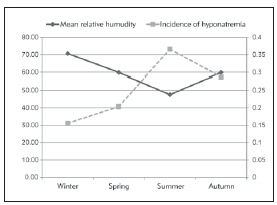
Incidence of hyponatremia (%) related to seasonal variability of relative humidity (%)

Mean temperature and relative humidity values of seasons in Isparta are presented in (Table 2). Hyponatremia incidence was found to be 15.4% in winter, 20.4% in spring, 28.7% in autumn, and 36.6% in summer. As a result of the comparison of the incidence of hyponatremia in the patients hospitalized in the winter and summer seasons, a signi ficantly higher incidence of hyponatremia was observed in the summer season (p<0.001). Although the mean serum sodium level was slightly higher in winter, the hyponatremia levels of the patients did not differ significantly between winter and summer (p=0.10). Besides, the incidence of hyponatremia in summer was significantly higher in elderly patients than in winter (24.8%, 7.09%, p<0.001, respectively). There was a positive correlation between seasonal mean temperature values and hyponatremia incidence (r=0.867, p=0.001, Figure 1). However, there was a negative correlation between seasonal mean relative humidity value and hyponatremia incidence (r=-0.735, p=0.001, Figure 2).

**Table 2:** Values of temperature and relative humidity according to the seasons Plus-minus values are mean ± standard deviation. ^a,b,c,d^ There is a significant difference between the parameters marked with the same letter (p<0.05).

		Autumn	Winter	Spring	Summer	p-value
Temperature (°C)	Mean	13.7±4.9^a^	3.4±2.5^a,b^	11.1±4.1^b,c^	22.7±2.3^a,c^	0.001
	Maximum	34.1	16.6	26.1	39.2	
	Minimum	-3.4	-12.9	-8.3	6.1	
Relative humidity (%)	Mean	59.9±8.9^a^	70.7±5.1^a,b^	59.9±3.8^b,c^	47.5±7.4^a,c^	0.001
	Maximum	96	97	94	81	
	Minimum	16	31	22	15	

There was no significant difference in the distribution of accompanying diseases and medications by the patients among seasons (p>0.05) (Table 1). Logistic regression analysis was conducted to determine the effect of medications, accompanying diseases, age, and gender, which might be linked to serum sodium level, in case of severe hyponatremia level (<125 mmol/L). The results of logistic regression analysis are presented in (Table 3). As only three patients had cirrhosis in summer and all hypertensive patients had chronic kidney disease (CKD), cirrhosis and hypertension were excluded from multivariate analysis. This multivariate analysis model had an accurate estimation ratio of 86.6%. There was an independent relationship between CKD and severe hyponatremia with an odds ratio of 2.69 (95% CI; 1.19-6.09), p=0.018. Two hundred and seven patients received hemodialysis treatment, and 28 patients received peritoneal dialysis treatment according to their renal replacement therapy requirements. A total of 96 patients received hemodialysis treatment, and 14 patients received peritoneal dialysis treatment in the summer. Additionally, 23 patients received hemodialysis, and 8 patients received peritoneal dialysis in winter. There was no difference between the two seasons in terms of the need for renal replacement therapy (p=0.17). An evaluation of hyponatremic patients regarding mortality revealed that 19 patients had died in a 4-year period. Of these patients, serum sodium levels of 12 were below 125 mmol/L. When the distribution of mortality among seasons was examined, it was found that 5 patients died in the summer, 6 patients in the autumn, 5 in the spring, and 3 in the winter. We found no difference among the seasons in terms of mortality (p=0.31). The volume status of the patients was shown in (Table 1). The ratio of hypervolemic patients in summer compared to those in winter and spring was significantly higher. However, there was no significant difference between summer and autumn.

**Table 3:** Variables associated with hyponatremia (<125 mmol/L) Abbreviations: CI, confidence interval; CKD, chronic kidney disease; AKI, acute kidney injury; CHF, congestive heart failure; ACEI, angiotensin-converting enzyme inhibitor; ARB, angiotensin receptor blocker; CCB, Calcium channel blocker.

		Odds ratio (95% CI)	*p* value
Age		1.0 (0.98–1.02)	0.75
Gender		0.91 (0.54–1.55)	0.72
CKD		2.69 (1.19–6.09)	0.018
AKI		1.91 (0.89–4.07)	0.09
CHF		0.89 (0.35–2.32)	0.82
Nephrotic syndrome		0.63 (0.15–2.59)	0.52
Diabetes mellitus		0.73 (0.41–1.30)	0.29
ACEI/ARB		1.19 (0.57–2.45)	0.65
CCB		0.51 (0.25–1.03)	0.06
Thiazide		1.18 (0.45–3.10)	0.74
Furosemide		1.51 (0.63–3.59)	0.36
Spironolactone		0.23 (0.05–1.10)	0.07
Antidepressant		1.88 (0.88–4.02)	0.10

## Discussion

In this single-centre observational study, we observed that there was a significant relation between hyponatremia incidence and seasons. Although serum sodium levels were slightly higher in winter when compared to those in summer, the difference was not statistically significant. In addition, we observed a positive correlation between seasonal temperature values and hyponatremia incidence and a negative correlation between seasonal relative humidity ratios and hyponatremia incidence.

In the causes of hyponatremia, extrarenal sodium losses such as diarrhoea, vomiting, pancreatitis; renal sodium losses caused by diuretic use, Addison's disease, salt-losing nephropathy; and increases in extracellular fluid volume such as congestive heart failure, cirrhosis, nephrotic syndrome, acute-chronic renal failure, inappropriate antidiuretic hormone secretion syndrome are the most common ones [11] [5]. Apart from these most common causes, researchers proposed that other factors might play a role in the development of hyponatremia. It has been stated that the weather condition is also an effective factor in the loss of water [6]. Spigset et al. [12] reported that hyponatremia related to antidepressant treatment was more common in psychiatric patients in summer. Chow et al. [10] determined that there was no relationship between hyponatremia caused by thiazide diuretics and seasonal change. Nicola et al. carried out a study on shipyard workers who received a low sodium diet and reported significantly higher numbers of hyponatremic patients in the summer months when compared to the winter months [13]. Although prior researches have shown varying results, Chakrapani et al. have found a relationship between hyponatremia incidence and seasonal change. They have shown that hyponatremia incidence increased in June-August, which was a period with high humidity and low-temperature values in the region where the study was conducted [9]. In the present study, we found higher hyponatremia incidence in summer when humidity value is low, and temperature value is high. All seasons considered, hyponatremia incidence was found to be higher in our study when compared to the value reported by Chakrapani et al. [9]. This difference may have arisen as a result of the inclusion of patients with serum sodium levels below 120 mmol/L in the study conducted by Chakrapani et al. [9]. In addition, it may have arisen as a result of the exclusion of the patients with medications that may affect sodium level and exclusion of the patients with additional diseases that may affect sodium level from the study. The incidence of hyponatremia was higher in elderly patients compared to adults in the study of Imai et al. [14]. Similarly, in this study, there was a higher incidence of hyponatremia in the elderly compared to adults. Likewise, a significant relationship was found between seasonal change and the incidence of hyponatremia in the elderly. This situation may be related to kidney failure and higher water intake in the elderly. During the 2003 heatwave in Lyon (France) and Barcelona (Spain), Argaud et al. [8] and Jimenez-Mejias et al. [15] reported hyponatremia incidence as 32% and 37%, respectively. During heat exposure, sweating occurs with an increase of less than 1 degree in body temperature [16]. It is known that environmental heat stress increases the sweating rate [17] [18] [19]. With the increase in sweating, there is an average loss of 4.8 to 6 grams of sodium equivalent to the salt loss of 10-15 grams [18]. These ratios are similar to hyponatremia incidence in the present study in summer (36.6%).

Hyponatremia related to polydipsia is generally considered as a dilutional condition as a result of fluid intake exceeding the excretory capacity of the kidneys [5]. Perspiration ratios of humans depend on various factors such as temperature, humidity, exercise and size of body surface area [20]. Godek et al. [19] reported that hyponatremia developed in football players as a result of fluid intake after exercise under hot weather conditions. The primary sweat passing through the sweat ducts on the body surface is almost similarly isotonic with blood plasma. Over time, sodium is reabsorbed via epithelial Na^+^ channels (ENaCs) and Na^+^/K^+^/ATPase channels. As a result, the last sweat thrown on the skin surface is hypotonic [21]. Afrai et al. stated in their study that there was an average loss of 6.8±2.1 gr NaCl with 1.5 litres of sweat after a yoga session [22]. In these cases, an appropriate amount of salt should be included in the lost water, or excessive hypotonic fluids should be avoided [22] [23]. In our study, higher hyponatremia incidence among inpatients in summer, when the temperature is higher than in winter, can have two explanations. The first one is excessive hypotonic fluid intake due to excessive perspiration by patients in the summer months when compared to winter. The second one is sodium loss through the skin by perspiration [18]. Hyponatremia can occur in hypervolemic euvolemic and hypovolemic conditions, and therefore volume assessment is important in determining the cause of hyponatraemia and management of treatment [24]. Tendency to hypervolemic hyponatremia increases due to excessive fluid intake in patients with renal failure [25]. Considering that the majority of individuals in the study population have CKD, the fact that the rate of hypervolemic patients is significantly higher in summer compared to winter can be seen as a supportive finding for our speculation about excessive hypotonic fluid intake as a result of sweating.

In this study, we found a negative relationship between relative humidity ratio and hyponatremia incidence. Insensible water loss is related to the humidity ratio of the environment, and inhalation of humid air can reduce insensible water loss approximately by 40% [9] [26]. In humid weather conditions, the decrease in the insensible fluid loss by air may lead to a relative decrease in hypotonic fluid intake. This may explain a significant decrease in the incidence of hyponatremia.

Chronic kidney disease is known to affect the capacity of kidneys that regulate water homeostasis, and therefore, an advanced CKD phase can increase the hyponatremia risk [27] [28]. Since the maximum dilution and concentration capacity of urine decrease during the course of CKD, the adaptation capacity of kidneys in excess water intake decreases and the tendency to hyponatremia increases [29]. There is a large body of research showing the relationship between hyponatremia and increased mortality [30]. Bennani et al. [31] have shown that <125 mmol/L serum sodium level in inpatients in intensive care unit was an independent risk factor in terms of mortality. Furthermore, a multivariate analysis conducted to investigate the effect of medications and diseases accompanying hyponatremia (serum sodium <125 mEq/L) has shown that CKD was a significant determinant factor in the development of severe hyponatremia. Considering the factors affecting hyponatremia, the fact that a great majority of the study group consisted of CKD patients might have caused this effect.

According to our knowledge, the results of this study are important as it is the first study showing the relationship between seasonal change and hyponatremia incidence in a set of inpatients in a nephrology clinic. However, the exclusion of emergencies (i.e., acute gastroenteritis, acute asthma, pandemic influenza, bacterial infections) and psychotic diseases that may affect hyponatremia are the limitations of this study, as well as being a small sample size and a retrospective study model.

## Conclusion

Based on the results of the study, it can be stated that increased temperature and decreased relative humidity are risk factors for a tendency towards hyponatremia. Especially for CKD patients, individualized management of fluid intake should be recommended rather than empirically designed fluid suggestions.

## Conflict of interest statement

All the authors declare that they have no conflict of interest in this work.
